# Pleomorphic Adenoma of Breast: Report of Two Cases, One in A Male Patient

**DOI:** 10.5146/tjpath.2021.01543

**Published:** 2022-05-19

**Authors:** Orwa Elaiwy, Khaled Al-Sawalmeh, Hayan Abo Samra, Abdulrazzaq Haider, Mohammed Akhtar

**Affiliations:** Department of Laboratory Medicine and Pathology, Hamad Medical Corporation, Doha, Qatar

**Keywords:** Pleomorphic adenoma, Breast, Male breast, Cartilage, Papilloma

## Abstract

Pleomorphic adenoma of breast (PAB) is a rare mammary tumor of a mixed epithelial-myoepithelial nature. We report two patients with PAB, one of which is male. We believe our male patient is the sixth case of PAB in male breast in the literature. The two cases expressed heterogeneous clinical and radiological characteristics while showing similar histology and immunohistochemical staining profile. The first case was managed with surgical resection while the second underwent interventional radiology excision. PAB is usually a benign entity with occasional cases of recurrence. Malignant transformation is rare but has been reported in a few cases.

## INTRODUCTION

Pleomorphic adenoma of breast (PAB) is a rare mammary tumor of a mixed epithelial-myoepithelial nature and is part of the so-called salivary gland-type mammary tumors, initially recognized in 1906. The existence of such tumors in the breast can be attributed to a shared embryonic origin between salivary and mammary glands (1). Although most patients are female with a wide age range, a few cases have also been reported in males. PABs typically manifest as a retro areolar, usually unifocal palpable mass (2). Imaging studies may show a fibroadenoma-like appearance that may harbor macrocalcifications (3). However, the rarity of these tumors makes it difficult to obtain an accurate radiological diagnosis prior to surgery; hence a lot of PABs are misdiagnosed preoperatively as carcinomas (4). Local excision is of both diagnostic and therapeutic importance since accurate diagnosis in many cases cannot be reached through needle core biopsy (5). PABs are grossly well demarcated, measuring on average 2 cm in maximum dimension and express a histological picture similar to pleomorphic adenoma of salivary gland consisting of cords and glandular formations of epithelial cells embedded in a chondro-myxoid stromal background (3). Most of the reported cases of PAB have behaved in a benign fashion; however, a few cases of malignant PAB were reported (5). Local recurrence has been reported after surgery; however, PABs show an overall favorable outcome (1). We report 2 cases of PABs in a 38-year-old male and a 79-year-old female patient. To the best of our knowledge, our first patient is the sixth case of PAB occurring in the male breast in the English literature.

## CASE REPORT

### Case 1

A 38-year-old man presented to the emergency department with a history of painful left breast swelling of 9 months duration with no nipple discharge. The swelling size had increased significantly during the last two days.

The physical examination revealed a hard swelling involving the left breast area, extending up to the nipple but not adherent to the skin or underlying chest wall.

**Figure 1:**
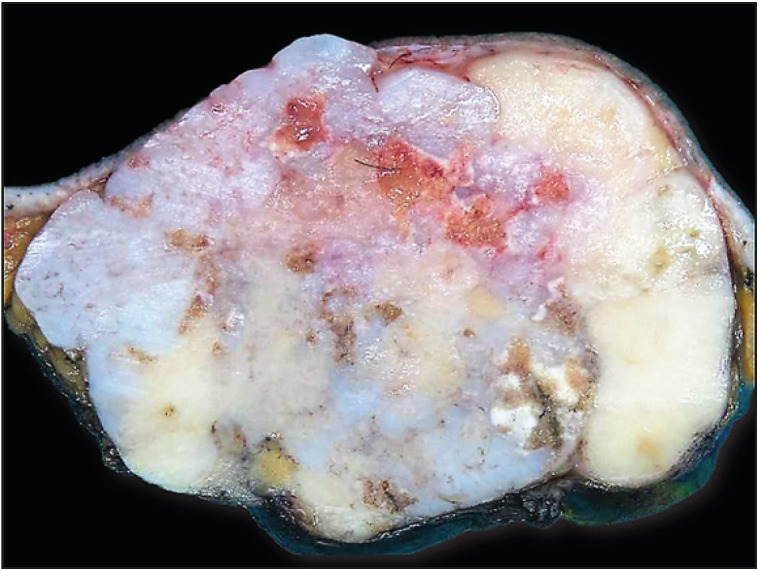
Case 1. A gross picture of pleomorphic adenoma of the breast (PAB) showing the yellow and tan-white calcified cut surface with prominent bluish white areas indicating cartilaginous differentiation.

**Figure 2:**
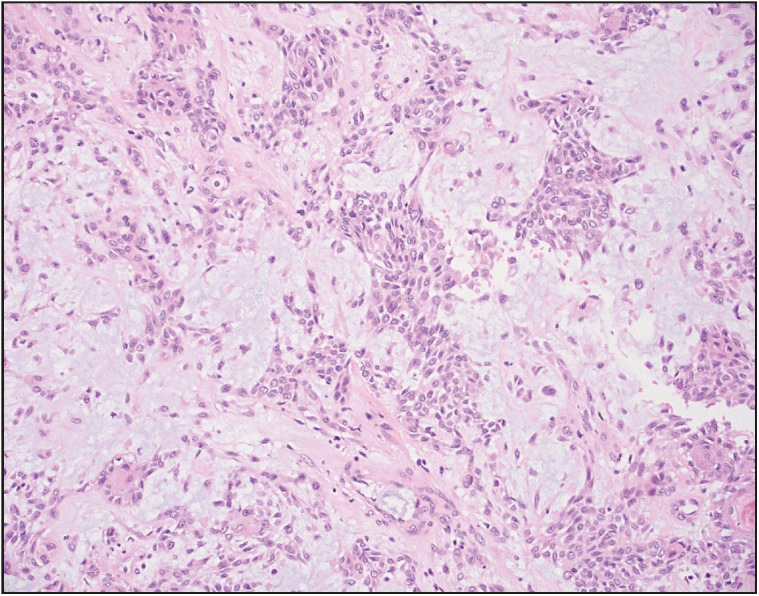
Case 1. Photomicrograph depicting glandular epithelial component in a background of myxoid stroma with prominent cartilaginous differentiation (Hematoxylin and Eosin stain, 100x).

**Figure 3:**
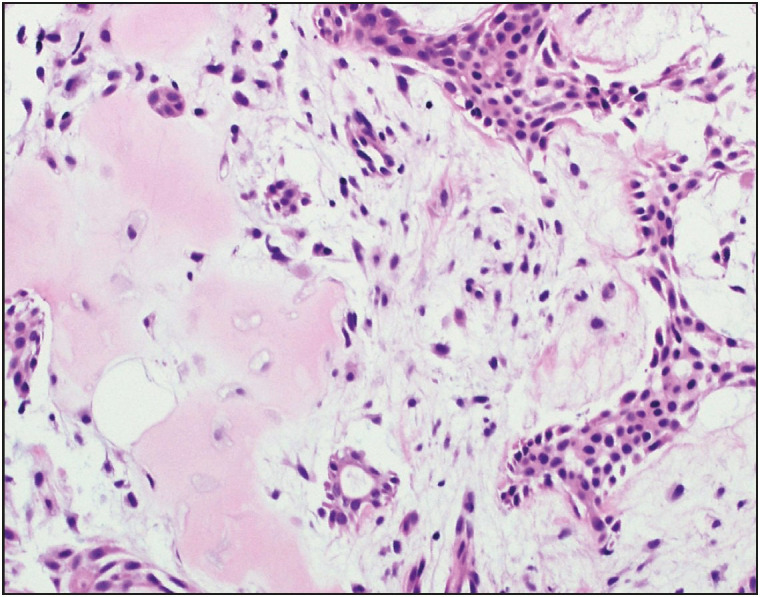
Case 1. Photomicrograph depicting glandular epithelial component in a background of myxoid stroma with prominent cartilaginous differentiation (Hematoxylin and Eosin stain, 200x).

MRI of the breast showed a large, bulging, and lobulated mass measuring 9.5 x 6.5 x 6 cm, located in the left breast with no associated skin edema/thickening. The mass abutted and partly compressed the underlying pectoral muscle without signs of muscle infiltration, and showed rim enhancement with washout and necrosis in the lateral aspect. The mass was given a score of BI-RADS 4 with rapidly growing phyllodes tumor cited as the main differential diagnosis. A core biopsy from the mass revealed mixed benign myoepithelial and glandular epithelial cells surrounded by chondromyxoid stroma with prominent cartilaginous differentiation. The cartilaginous areas showed no features of atypia, and neither did the epithelial parts, which ruled out a possible differential diagnosis of metaplastic carcinoma. By immunohistochemistry the myoepithelial cells expressed Smooth Muscle Myosin, p63 and CK5/6; glandular epithelial cells expressed CKAE1/AE3; stromal cells expressed GFAP; and cartilaginous cells in stroma expressed S100. Estrogen Receptor (ER) and Smooth Muscle Actin (SMA) were negative. The subsequent excision specimen contained a lobulated mass measuring 9 x 8 x 3 cm with a partially red to yellow and partially tan-white calcified cut surface. Several areas revealed bluish-white coloration indicating cartilaginous differentiation (Figure 1). Microscopically this mass showed identical histology to the biopsy (Figure 2, Figure 3); no immunohistochemistry was performed on the excision specimen and the diagnosis remained the same in both specimens as pleomorphic adenoma of the breast.

### Case 2

A 79-year-old woman presented to the breast screening clinic for the annual mammography which showed a small well-defined sub areolar nodule in the right breast. An ultrasonography revealed a complex cystic lesion at 7 o’clock and sub areolar region, measuring 1 x 0.6 cm. The lesion was well-defined and had intra cystic nodular component with mild internal vascularity.

**Figure 4:**
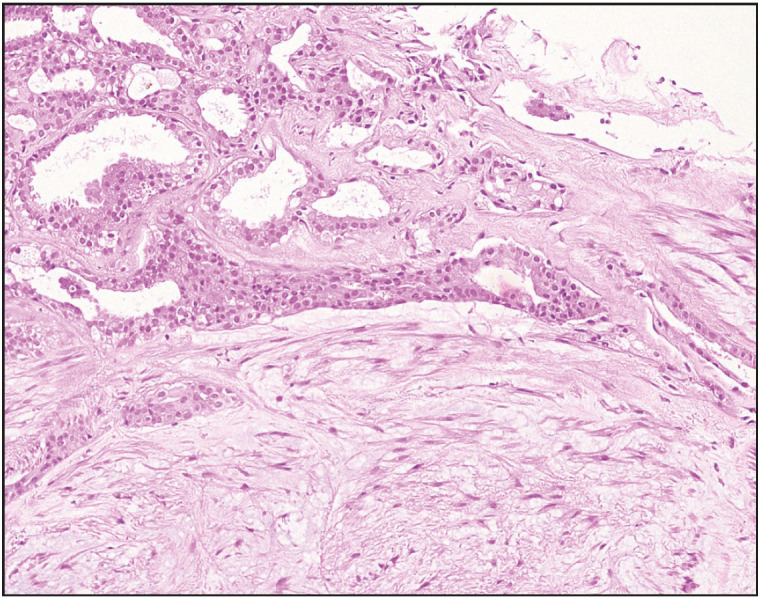
Case 2. Photomicrograph showing PAB’s glandular component in a chondromyxoid stroma (Hematoxylin and Eosin stain, 100x).

**Figure 5:**
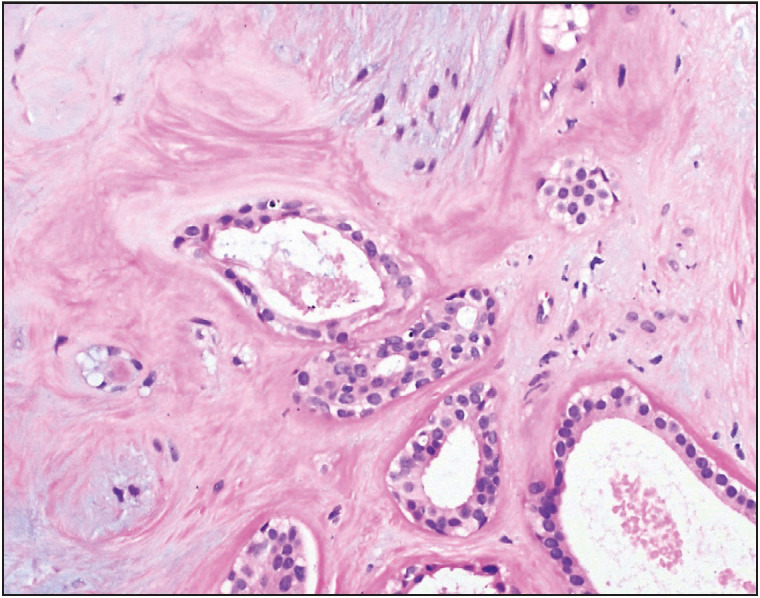
Case 2. Photomicrograph showing PAB’s glandular component in a chondromyxoid stroma (Hematoxylin and Eosin stain, 200x).

**Figure 6:**
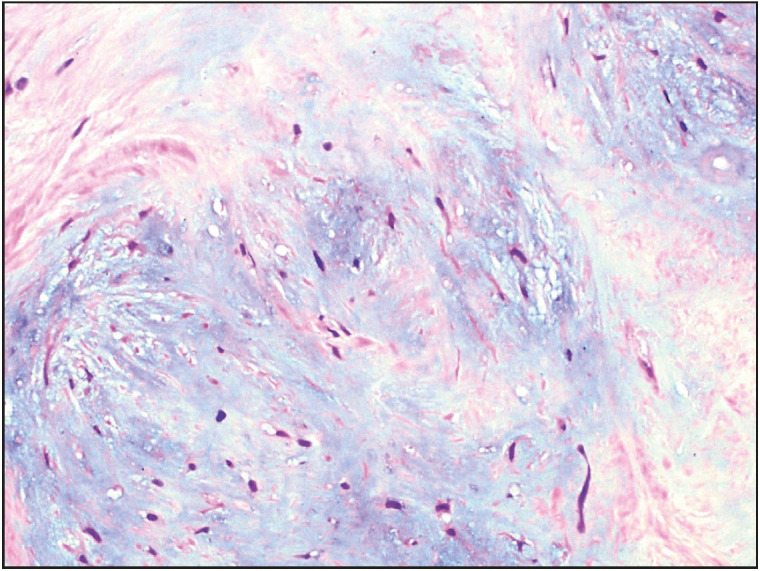
Case 2. Photomicrograph depicting prominent myxoid differentiation of the stroma (Hematoxylin and Eosin stain, 200x).

**Figure 7:**
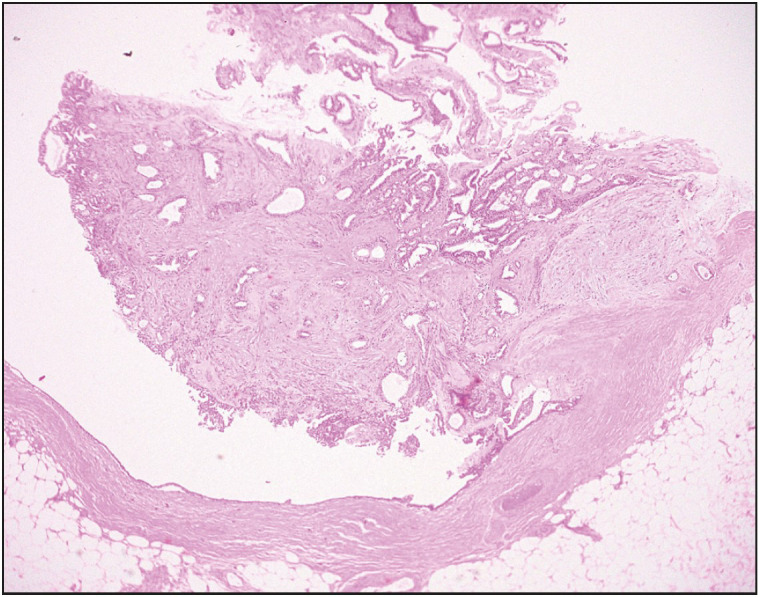
Case 2. Photomicrograph depicting PAB in the right side of the picture with adjacent intraductal papilloma in the left side (Hematoxylin and Eosin stain, 40x).

**Figure 8:**
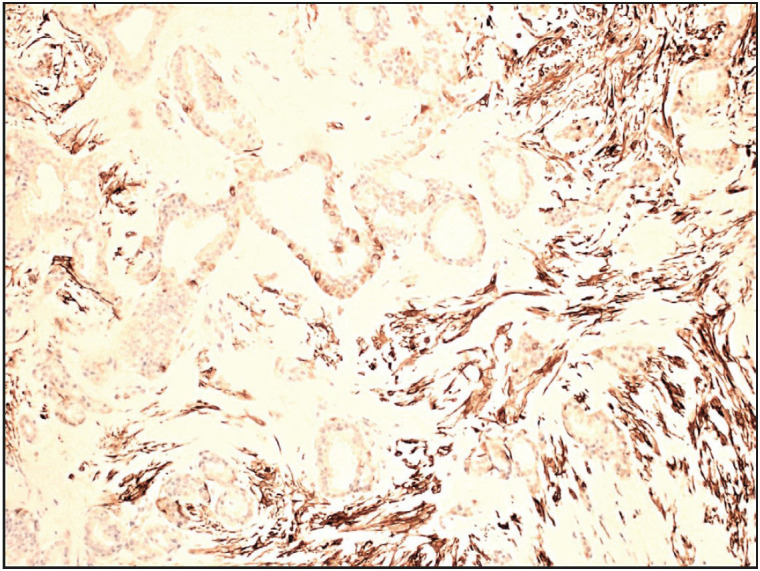
Case 2. Photomicrograph depicting immunohisto-chemical staining of stromal cells for GFAP (Immunohistochemical stain, 100x).

A vacuum-assisted excisional biopsy yielded multiple fibroadipose fragments measuring 2.7 x 2.3 x 0.5 cm in aggregate. Microscopic examination showed a part of a circumscribed nodule harboring benign glands and tubules composed of glandular epithelium along with myoepithelial cells in a background of variably fibrotic and chondromyxoid stroma (Figure 4, Figure 5, Figure 6), as well as an intraductal papilloma component in the vicinity (Figure 7). Although the presence of chondromyxoid stroma might have suggested the differential diagnosis of metaplastic carcinoma, the lack of atypia or even hypercellularity in both epithelial and stromal components negated that differential. Immunohistochemistry showed expression of Smooth Muscle Myosin, p63, S-100, GFAP and CK 5/6 in myoepithelial cells while glandular epithelial cells expressed CK 7 (Figure 8). A diagnosis of pleomorphic adenoma of breast with associated intraductal papilloma was established. The patient had no further interventions. During a follow up hospital visit more than a year later, the patient had no breast-related complaints.

## DISCUSSION

Although Pleomorphic Adenoma (PA) is a relatively common neoplasm in the salivary glands, its mammary counterpart is extremely rare with less than 80 cases reported since their first description in 1906 (2,6). PAB patients show a wide age range from third to eighth decades, with the majority in the post-menopausal age (1), a feature expressed by our second patient. Only five cases have been reported in the male breast in the literature (6–10) with one tumor arising in an accessory nipple (11). In the published literature so far, our first patient is the sixth case of PAB in a male patient. Table 1 summarizes all the reported cases of PABs in male patients to date including one tumor arising in an accessory nipple.

**Table 1:** Reported cases of breast and nipple pleomorphic adenomas in male patients.

**Case number**	**Author(s)**	**Age (years)**	**Anatomical site and laterality**	**Tumor Size (cm)**
1	Gioia and Bianchi (1930) (6)	42	Right breast, superio-medial quadrant	4
2	Makek and Von Hochstetter (1980) (7)	60	Right breast, superio-medial quadrant	1.5
3	Simha et al. (1992) (8)	65	N/A	N/A
4	Butrón et al. (1997) (9)	40	Left breast	3.5
5	Molland et al. (2005) (10)	29	Left breast, retroareolar	1.8
6	Scaranelo (2019) (11)	40	Right chest wall, accessory nipple	7
7	Present case	38	Left breast, retroareolar	9

PABs are among the so-called salivary gland-type mammary tumors (1), a heterogeneous group of tumors that are usually subclassified into epithelial, myoepithelial and epithelial-myoepithelial morphologic subtypes (i.e., biphasic); the last of which includes PABs among others (3). The majority manifest clinically as a palpated retroareolar solitary lump (2). Diagnostic imaging findings varied significantly among authors, with some reporting fibroadenoma-like appearance that may harbor macrocalcifications (3), while others reported features ranging from completely calcified fibroadenomas to those suggesting carcinomas and mucinous carcinomas (4). Our patients also manifested this variety, with the first patient having a palpable mass expressing radiologic features of phyllodes tumor; while the second patient had asymptomatic lesion that was only described by radiology as a complex cyst. The rarity of PABs in the literature has made it extremely difficult to establish a diagnosis even on core biopsies, with a lot of cases being misdiagnosed as carcinoma (4). The differential diagnosis for PABs is a wide list, with metaplastic carcinomas at the top of the list as the most worrisome possibility. The latter, along with matrix-producing carcinomas must all be excluded, a challenging task in core biopsies. While the presence of unequivocal features of malignancy (like severe nuclear atypia, absence of myoepithelial cells and abundant mitotic activity among others) favors the diagnosis of carcinoma, the absence of those features in core biopsies does not negate malignancy elsewhere in the lesion as many cases were found to be malignant only after excision and examining the entire lesion (1,3).

This has made surgery the mainstay of diagnosis and management, with local excision and subsequent histological examination of the specimen serving as the best way to reach an accurate diagnosis (5). Gross examination of PAB usually yields a well-demarcated lesion that measures around 2 cm in maximum dimension (3). Microscopically PABs resemble salivary gland pleomorphic adenomas with a cellular component composed of cords and glandular formations of epithelial and myoepithelial cells embedded in a variable chondro-myxoid stromal component (3). An intraductal papilloma is often seen in the vicinity of PAB, which led many authors to consider them a variant of papilloma, while other authors associated PABs with other benign neoplasms like ductal adenomas (1). This feature was expressed in the PAB of our second patient, which featured a microscopic intraductal papilloma component. Immunohistochemically, PABs express SMA and S100 and GFAP in the myoepithelial cells and EMA and cytokeratin in the epithelial cells (5) while being negative for ER, PR and Her-2 (triple-negative) (1). Salivary pleomorphic adenomas express translocations in chromosomes affecting genes *PLAG1*, *HMGI-C* and *HMGIY*. Immunohistochemical expression of HMGI-C and HMGIY have been reported, but more research studying the genetics of PABs is needed (2).

PABs express mainly a benign clinical course, even with reports of cases recurring locally (3). Carcinoma ex pleomorphic adenoma is the term describing malignant transformation of PAB, an extremely exceptional complication that has only been reported three times before (3). Because of this slim malignant transformation potential, some authors proposed annual follow up extending to five years in PAB cases.

## CONCLUSION

We reported two cases of PAB, one of which occurred in a male patient. The diagnostic work up for these cases took a significant effort clinically, radiologically and pathologically. Both cases were managed with excision. Although the first case had a wide surgical excision, the second was managed through interventional radiology. The rarity of this entity, as well as the potential for misdiagnosis makes it imperative to keep this possibility in mind while working up a breast mass lesion.

## Conflict of Interest

The authors declare no conflict of interest.
